# Fabrication of Poly(s-triazine-*co*-*o*-aminophenol) Conducting Polymer via Electropolymerization and Its Application in Aqueous Charge Storage

**DOI:** 10.3390/polym17091160

**Published:** 2025-04-24

**Authors:** Xueting Bai, Bo Lan, Xinyang Li, Xinlan Yi, Shaotong Pei, Chao Wang

**Affiliations:** 1 Hebei Provincial Key Laboratory of Power Transmission Equipment Security Defense, North China Electric Power University, Baoding 071003, China; xt_baii@163.com (X.B.); rambo596596@163.com (B.L.); lxygerenyouxiang@163.com (X.L.); peishaotong@ncepu.edu.cn (S.P.); 2 Economic Management Department, North China Electric Power University, Baoding 071003, China; yixinlan1006@163.com; 3 School of Renewable Energy, Inner Mongolia University of Technology, Ordos 017010, China; 4 Inner Mongolia Key Laboratory of New Energy and Energy Storage Technology, Hohhot 010051, China

**Keywords:** conducting polymer, PT-*co*-oAP, electropolymerization, aqueous electrolyte, electrochemical energy storage

## Abstract

Designing conducting polymers with novel structures is essential for electrochemical energy storage devices. Here, copolymers of s-triazine and o-aminophenol are electropolymerized from an aqueous solution onto a carbon cloth substrate using the galvanostatic method. The poly(s-triazine-*co*-*o*-aminophenol) (PT-*co*-oAP) is characterized, and its charge storage properties are investigated in 1 M H_2_SO_4_ and in 1 M ZnSO_4_. At 1 A g^−1^, the specific capacities of PT-*co*-oAP reach 101.3 mAh g^−1^ and 84.4 mAh g^−1^ in 1 M H_2_SO_4_ and in 1 M ZnSO_4_, respectively. The specific capacity of PT-*co*-oAP maintains 90.3% of its initial value after cycling at 10 A g^−1^ for 2000 cycles in 1 M H_2_SO_4_. The high specific capacity achieved originates from abundant surface active sites, facile ion diffusion, with optimized active site structure achieved by forming copolymer. The charge storage mechanism involves the redox processes of amino/imino groups and hydroxyl/carbonyl groups in the copolymer, together with the insertion of cations. Two electrode devices using two PT-*co*-oAP and aqueous 1 M H_2_SO_4_ are assembled, and the maximum energy density reaches 63 Wh kg^−1^ at 0.5 A g^−1^ with a power density of 540 W kg^−1^. The capacity retention of the device after 3000 cycles at 10 A g^−1^ reaches 81.2%.

## 1. Introduction

Electrode materials are essential for high-performance energy storage devices. Conducting polymers, including polyaniline and polypyrrole [1,2], are environmentally benign, conductive, and inexpensive, and they are considered promising electrode materials for charge storage [3]. However, due to the repetitive ion insertion and extraction during charging and discharging, as the electrode material, the stability of conducting polymers needs improvement [4]. Therefore, designing new conductive polymers with novel morphologies and novel chemical structures is a promising route to achieve high specific capacity and stability [5,6,7].

S-triazine-based covalent bonded frameworks were recently reported to exhibit excellent charge storage capacity [8]. S-triazine derivatives with aromatic rings exhibit good thermal stability and a conjugated D-π-A structure, which facilitates charge separation and charge transport. The s-triazine ring is capable of binding with cations via π interactions and with anions through σ interactions. In this way, abundant charge can be stored, endowing s-triazine a promising monomer to construct conducting polymers [9]. *O*-aminophenol contains amino and phenolic hydroxyl groups and is also reported to act as a monomer to construct conducting polymers for charge storage [10]. In poly(*o*-aminophenol) (PoAP), hydroxyl/carbonyl and amino/imino groups can serve as active sites to store charge. Graphene nanosheets-PoAP nanocomposites prepared by potential cycling on a platinum surface by Heli et al. exhibit a specific capacitance of 281.1 F g^−1^ at 0.1 A g^−1^ [11]. Functionalized graphene oxide nanosheets/PoAP were prepared by Ehsani et al., and the specific capacitance reached 251.15 F g^−1^ [12]. Maryam Naseri et al. used electropolymerization to prepare PoAP/ZnO, which exhibits a specific capacitance of 223 F g^−1^ [13]. Depending on the electrochemical method adopted, different structures of poly(*o*-aminophenol), including line-shaped and ladder-shaped repeating units, can be constructed.

Electropolymerization offers a facile route to construct copolymers, either layer-by-layer or random copolymers [14,15]. Copolymers exhibit unique charge storage properties by combining the functional groups of two monomers. For example, poly(5-amino-naphthalene sulfonic acid-*co*-*o*-aminophenol) (PANS-*co*-oAP) was electropolymerized on a carbon cloth (CC) substrate in an aqueous acid electrolyte with both monomers, exhibiting superior charge storage performance compared to PANS and PoAP [16]. The simultaneous presence of electron-donating (amino and hydroxyl) and electron-absorbing (sulfonic acid) groups in copolymers facilitates charge separation and the generation of active sites. S-triazine is electron-deficient, and constructing copolymers with electron-rich monomers is desirable to achieve efficient charge separation and the generation of active sites [17]. Therefore, the copolymer of s-triazine and oAP is constructed using electropolymerization. The copolymer is characterized, and its charge storage properties are investigated.

## 2. Experimental

The following reagents are used without further purification: 1,3,5-triazine (C_3_H_3_N_3_, AR, 97%, Shanghai BiDe Pharmaceutical Technology Co., Ltd., Shanghai, China), *o*-aminophenol (C_6_H_7_NO, AR, 99%, Shanghai Aladdin Biochemical Technology Co., Ltd., Shanghai, China), concentrated sulfuric acid (H_2_SO_4_, AR, 98.0%, Shanghai HaoHong Bio-Pharmaceutical Technology Co., Ltd., Shanghai, China), zinc sulfate (ZnSO_4_, AR, 99.8%, Shanghai HaoHong Bio-Pharmaceutical Technology Co., Ltd., Shanghai, China), carbon cloth (SCC130, Suzhou ShengErNuo Technology Co., Ltd., Suzhou, China), and distilled water.

Electropolymerization was used to obtain copolymers with CC as the substrate in aqueous acidic solution. The CC was first cut into small pieces of 1 × 2 cm^2^, washed repeatedly with water for 30 min, and calcined at 400 °C for 90 min. When used as the working electrode, about a 1 × 1 cm^2^ surface area was immersed in the electrolyte. Electropolymerization was carried out using the galvanostatic method, which can deposit measurable amounts of polymer on CC in a short time. A cleaned graphite rod was used as the counter electrode, and a saturated calomel electrode (SCE) was used as the reference in the three-electrode system setup. The electrolyte was a 1 M H_2_SO_4_ aqueous solution containing 5 mM s-triazine and 2 mM oAP. A constant current density of 0.01 A cm^−2^ was applied to construct PT-*co*-oAP. The mass of the PT-*co*-oAP film obtained by electropolymerization on CC was measured to be 1.0 mg cm^−2^ using a laboratory balance. The PT and PoAP were prepared using a similar method, with the electrolyte containing only 5 mM s-triazine or 2 mM oAP, respectively, and the deposited masses were 0.4 mg cm^−2^ and PoAP 0.3 mg cm^−2^, respectively. Information about instrumentation and equations is provided in the Supporting Information.

## 3. Results and Discussion

**Figure 1:**
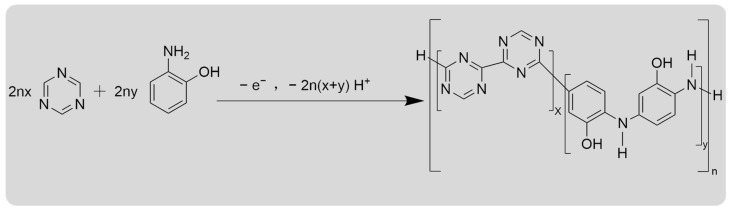
Proposed structure of PT-*co*-oAP.

**Figure 2:**
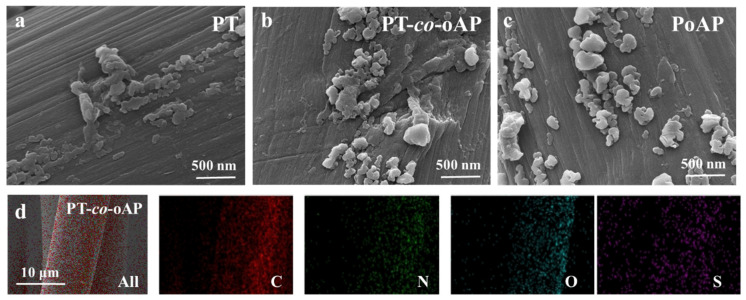
SEM images of (**a**) PT, (**b**) PT-*co*-oAP, and (**c**) PoAP; (**d**) EDS elemental mapping of PT-*co*-oAP.

Electropolymerization using the galvanostatic method enables a controlled electropolymerization rate to be achieved, and large quantities of polymers can be generated in a short period [18,19]. The PT-*co*-oAP, PT, and PoAP were electrochemically polymerized onto a CC substrate from 1 M H_2_SO_4_ (Figure S1, Supporting Information). Electropolymerization is initiated by the generation of radicals at the N sites in oAP and s-triazine. The radicals can attack adjacent neutral monomers or couple with another radical to form oligomers. With the repetitive generation of radicals and the growth of the oligomer chain, deposition happens when the concentration of the oligomer near the electrode surface exceeds solubility [20]. Solid-state radical generation and coupling form the three-dimensional connected film at the electrode surface. Figure 1 shows the proposed structure of the PT-*co*-oAP film. Figure 2a–c show the scanning electron micrographs (SEM) of PT, PT-*co*-oAP, and PoAP, and the SEM of the bare CC is provided in Figure S5. In Figure 2a, the CC surface is covered by a layer of PT, and the surface appears rough. In Figure 2b, the PT-*co*-oAP exhibits a small granular structure of about 300 nm in size. Figure 2c displays the SEM of PoAP, which also exhibits a granular structure. Figure 2d shows the electron diffraction spectroscopy (EDS) elemental mapping of PT-*co*-oAP. The N is from both PT and oAP, and the uniform distribution of N and O indicates the successful formation of PT-*co*-oAP on CC. Note that small amounts of S are also detected from the PT-*co*-oAP, which result from the intercalated anions (SO_4_^2−^) during electropolymerization to balance the positive charge of the polymer [21].

**Figure 3:**
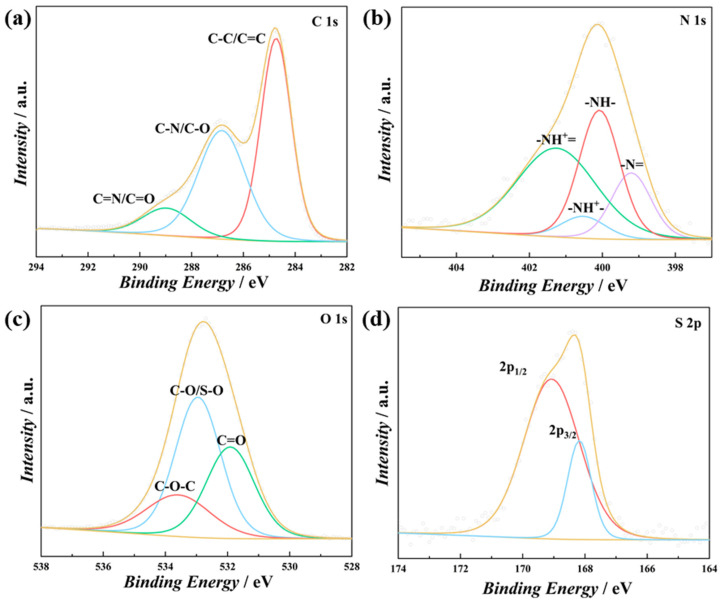
XPS spectra of (**a**) C 1s, (**b**) N 1s, (**c**) O 1s, and (**d**) S 2p regions of PT-*co*-oAP.

The surface state of the deposited PT-*co*-oAP was investigated by X-ray photoelectron spectroscopy (XPS, Figure 3). Figure 3a shows the deconvoluted high-resolution XPS spectra of the C 1s region of PT-*co*-oAP. The deconvolved peaks at 284.7, 286.8, and 289.0 eV correspond to the C-C/C=C, C-N/C-O, and C=N/C=O bonds, respectively [22]. The N 1s spectra (Figure 3b) can be deconvoluted into 399.2, 400.1, 400.5, and 401.3 eV peaks assignable to neutral amine (-NH-), neutral imine (-N=), protonated amine (-NH^+^-), and protonated imine (-NH^+^=), respectively [23]. The doping level calculated by analyzing the ratio of protonated amino and imino groups to the total N content is 0.51, which is a typical doping level for conducting polymers [22,24]. This indicates that approximately half of the N is protonated. Figure 3c shows the deconvoluted high-resolution XPS spectra of the O 1s region, with peaks at 531.9, 533.0, and 533.6 eV, corresponding to C=O, C-O/S-O, and C-O-C bonds, respectively. The C=O bonds originate from the carbonyl groups formed by the oxidation of the hydroxyl groups on the oAP or the carbonyl groups generated during the oxidative electropolymerization of s-triazine [9,16]. Figure 3d shows the XPS deconvoluted spectra of the S 2p region, with peaks at 169.1 (2p_1/2_) and 168.2 eV (2p_3/2_) corresponding to S in the intercalated sulfate groups [25]. These characterizations show that the PT-*co*-oAP film is successfully electropolymerized onto the CC surface.

**Figure 4:**
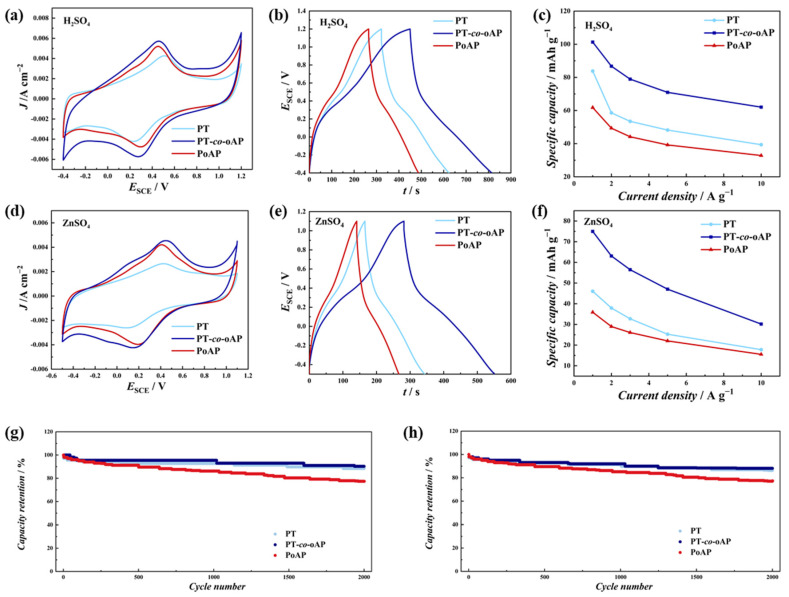
(**a**) CV of PT, PoAP, and PT-*co*-oAP from −0.4 to 1.2 V_SCE_ at scan rate of 20 mV s^−1^ in 1 M H_2_SO_4_; (**b**) GCD curves of these electrodes at 1 A g^−1^ charge and discharge current density in 1 M H_2_SO_4_; (**c**) GCD specific capacities versus current densities in 1 M H_2_SO_4_; (**d**) CV of these electrodes from −0.5 to 1.1 V_SCE_ at scan rate of 20 mV s^−1^ in 1 M ZnSO_4_; (**e**) GCD curves at 1 A g^−1^ in 1 M ZnSO_4_; (**f**) GCD specific capacities versus current densities in 1 M ZnSO_4_. Cycle stability of these electrodes at 10 A g^−1^ in (**g**) 1 M H_2_SO_4_ and in (**h**) 1 M ZnSO_4_.

Three electrode tests were carried out to investigate the charge storage properties of PT, PoAP, and PT-*co*-oAP in 1 M H_2_SO_4_. The cyclic voltammetry (CV) of PT, PoAP, and PT-*co*-oAP in 1 M H_2_SO_4_ in the range of −0.4–1.2 V_SCE_ at 20 mV s^−1^ are shown in Figure 4a. For the PT, the redox peaks are at 0.51 V_SCE_ (ox.) and 0.23 V_SCE_ (red.), which is consistent with reference [9]. For the PoAP, two pairs of redox peaks are present, and the redox peaks at 0.45 V_SCE_ (ox.)/0.3 V_SCE_ (red.) are significantly higher than those at 0.2 V_SCE_ (ox.)/0.1 V_SCE_ (red.). This indicates that the linear structure is predominant in PoAP, not the ladder structure, and that phenoxazine units are predominant. The redox peaks of PT-*co*-oAP are at 0.46 V_SCE_ (ox.)/0.27 V_SCE_ (red.), which are in the middle of PoAP and PT. This implies that a random copolymer is formed, and the redox properties are tuned through the electron interaction between the s-triazine and oAP units. The redox peaks originate from the redox of hydroxyl/carbonyl groups and amino/imino groups, accompanied with cation insertion and extraction. The GCD curves of PT, PoAP, and PT-*co*-oAP (Figure 4b) in 1 M H_2_SO_4_ at 1 A g^−1^ all show a small plateau around 0.46 V_SCE_. The GCD curves of PT and PoAP are consistent with the CV of PT and PoAP. The potential of this plateau matches the redox peak potential observed in CV. The specific capacities of PT, PoAP, and PT-*co*-oAP in 1 M H_2_SO_4_ are calculated by GCD to be 83.8, 62.8, and 101.3 mAh g^−1^, respectively. The PT-*co*-oAP exhibits higher specific capacity than PT and PoAP. The specific capacities of PT, PoAP, and PT-*co*-oAP at different current densities are shown in Figure 4c. When discharged at 2, 3, 5, and 10 A g^−1^, the calculated specific capacities of PT-*co*-oAP are 93.3, 90.8, 87.8, and 84.4 mAh g^−1^, respectively. The decrease in specific capacity with increasing current density is due to the slow kinetics of the redox process limited by ion insertion and extraction [26,27]. The capacity retention values of PT, PoAP, and PT-*co*-oAP are 47.0%, 53.2%, and 61.2%, respectively, when the GCD current density changes from 1 to 10 A g^−1^. PT-*co*-oAP exhibits the highest capacity retention among these electrodes.

As increasing attention is being paid to energy storage systems in aqueous electrolytes with zinc ions, zinc sulfate solution was used as the electrolyte to evaluate the charge storage properties [28,29]. The CV values of PT, PoAP, and PT-*co*-oAP in 1 M ZnSO_4_ are given in Figure 4a. The redox peaks of PT are located at 0.42 V_SCE_ (ox.)/0.09 V_SCE_ (red.), while those of PoAP are located at 0.41 V_SCE_ (ox.)/0.19 V_SCE_ (red.). The PT-*co*-oAP exhibits redox peaks at 0.45 V_SCE_/0.15 V_SCE_. These redox peaks also originate from the redox processes of hydroxyl/carbonyl groups and amino/imino groups, accompanied with cation insertion and extraction. The GCD curves of these electrodes at 1 A g^−1^ are shown in Figure 4e. The specific capacities of PT, PoAP, and PT-*co*-oAP are 46.0, 35.9, and 75.0 mAh g^−1^, respectively. When discharged at 2, 3, 5, and 10 A g^−1^, the specific capacities of PT-*co*-oAP are 63.1, 56.4, 47.0, and 30.2 mAh g^−1^, respectively (Figure 4f). When the GCD current density is in the range of 1 to 10 A g^−1^, the capacity retention values of PT, PoAP, and PT-*co*-oAP are 38.6%, 43.3% and 40.3%, respectively, and significantly lower capacity retention is observed in 1 M ZnSO_4_ compared to 1 M H_2_SO_4_, which might result from the complex side reactions in 1 M ZnSO_4_ that limit the kinetics at high charge and discharge rates [30,31,32]. The cycling stability of these electrodes in 1 M H_2_SO_4_ and 1 M ZnSO_4_ at 10 A g^−1^ is also investigated, and the results are shown in Figure 4g,h. After 2000 cycles, the specific capacity retention values of PT, PoAP, and PT-*co*-oAP are 88.4%, 77.4%, and 90.3% in 1 M H_2_SO_4_, respectively, and 86.3%, 77.1%, and 88.1% in 1 M ZnSO_4_, respectively. This indicates that PT-*co*-oAP exhibits the best cycling stability in both electrolytes. In addition, PT-*co*-oAP also sustains the 10,000-cycle stability test (Figure S4). SEM and XPS after the stability test (Figures S6 and S9) indicate no significant physical detachment of the PT-*co*-oAP from the CC substrate. This implies that specific capacity degradation is induced from the structural change that leads to a loss of active sites.

**Figure 5:**
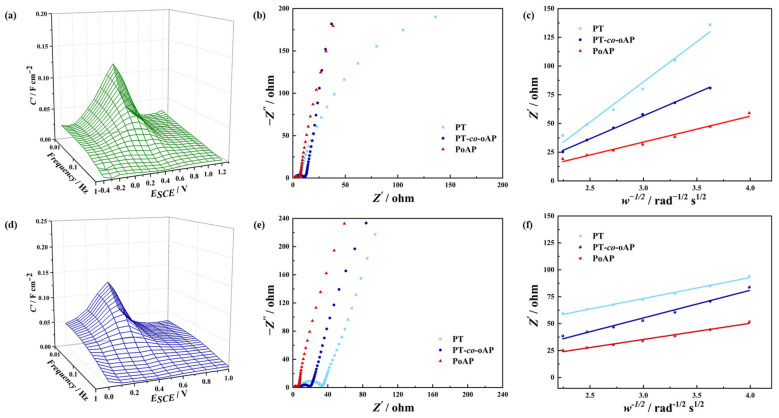
(**a**) Three-dimensional Bode plots of C’ vs. f vs. E of PT-*co*-oAP in 1 M H_2_SO_4_. (**b**) Nyquist plots of PT-*co*-oAP in 1 M H_2_SO_4_. (**c**) Plot of Z’ vs. ω^−1/2^ of PT-*co*-oAP in 1 M H_2_SO_4_. (**d**) Three-dimensional Bode plots of PT-*co*-oAP in 1 M ZnSO_4_. (**e**) Nyquist plots of PT-*co*-oAP in 1 M ZnSO_4_. (**f**) Plot of Z’ vs. ω^−1/2^ of PT-*co*-oAP in 1 M ZnSO_4_.

Electrochemical impedance spectroscopy was then carried out [33]. Figure 5a shows the 3D Bode plot of PT-*co*-oAP obtained in 1 M H_2_SO_4_ [34]. The 3D Bode plot shows a low frequency peak at −0.2–0.8 V_SCE_, which is consistent with the redox peak potential in CV. Together with the redox peaks in CV and the asymmetric shape of the GCD curves, the peak in the 3D Bode plot implies that the PT-*co*-oAP is a battery-type charge storage material. The Nyquist plots of these electrodes are shown in Figure 5b. The high-frequency semicircles of the Nyquist plots of PT, PoAP, and PT-*co*-oAP can be observed, whose diameter is related to the charge transfer resistance (*R*_CT_) [35]. The *R*_CT_ of PT-*co*-oAP is the largest, which indicates that the redox kinetics of PT-*co*-oAP is the most sluggish. The low-frequency region *Z*’ versus *ω*^−1/2^ in 1 M H_2_SO_4_ is shown in Figure 5c. The slope ∂ (Warburg factor) is related to ion diffusion impedance [36,37]. ∂ is the smallest for PT-*co*-oAP (40 Ω s^−1/2^ rad^1/2^), which indicates that ion diffusion is the most facile for PT-*co*-oAP. The ∂ values are 70 and 23 Ω s^−1/2^ rad^1/2^ for PT and PoAP, respectively. Figure 5d exhibits the 3D Bode plot for PT-*co*-oAP in 1 M ZnSO_4_, with low-frequency peaks in the range of −0.2–0.7 V_SCE_. PT-*co*-oAP behaves as a battery-type electrode material in 1 M ZnSO_4_. The Nyquist plots of PT, PoAP, and PT-*co*-oAP in 1 M ZnSO_4_ (Figure 5e) are typical for capacitive behavior, where a straight line close to the −*Z*″ axis is shown. The *R*_CT_ of PT-*co*-oAP is lower than that of PT but higher than that of PoAP. In both electrolytes, the *R*_CT_ of PoAP is the lowest, which indicates its most facile redox kinetics. The ∂ values of PT, PoAP, and PT-*co*-oAP are 20, 15, and 26 Ω s^−1/2^ rad^1/2^, respectively. In both electrolytes, PT exhibits the highest diffusion impedance, which is probably a result of the thick-layered morphology of PT. In contrast, PT-*co*-oAP exhibits the most facile diffusion, probably originating from the porous granular shape of the deposits. Also, the change in interfacial hydrogen bonding structure can affect the ion diffusion processes. However, the *R*_CT_ of PT-*co*-oAP is not the lowest, which implies that the redox kinetics is not accelerated, which is possibly limited by the electron transfer process induced by the formation of copolymers. In addition, the structure of the active sites is altered, and the electron interaction may also affect the redox kinetics. From the CV, PT-*co*-oAP exhibits the largest integrated area, which indicates that the number of active sites is the highest. Though redox kinetics is not the most facile, the abundant number of electrochemically active sites and facile diffusion enhance the charge storage performance of PT-*co*-oAP. The SEM images show that these electrodeposited films consist of granular and aggregated structures. Though this morphology is common for electrodeposited films, a highly porous substrate is desirable for polymer deposition to expose a higher number of electrochemically active sites for charge storage.

**Figure 6:**
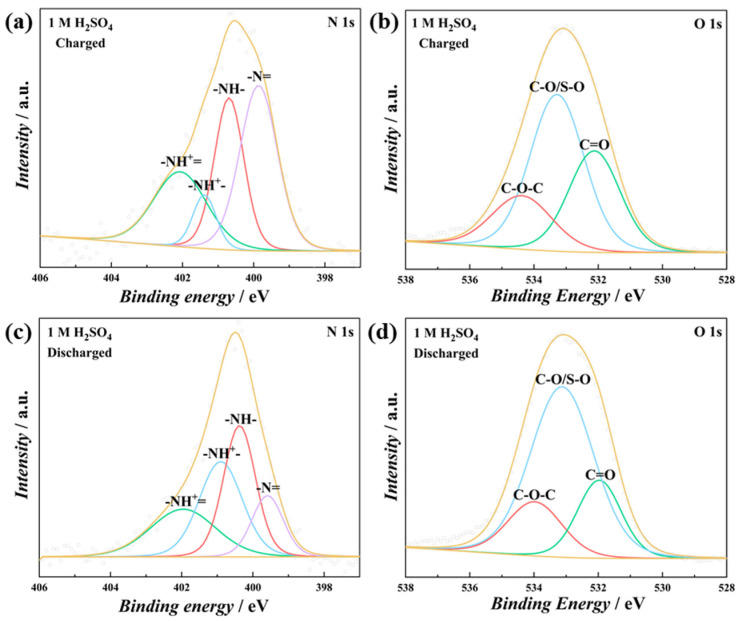
Deconvoluted XPS spectra of PT-*co*-oAP. (**a**) N 1s region and (**b**) O 1s region when charged to 1.2 V; (**c**) N 1s region and (**d**) O 1s region when discharged to −0.4 V in 1 M H_2_SO_4_.

**Figure 7:**
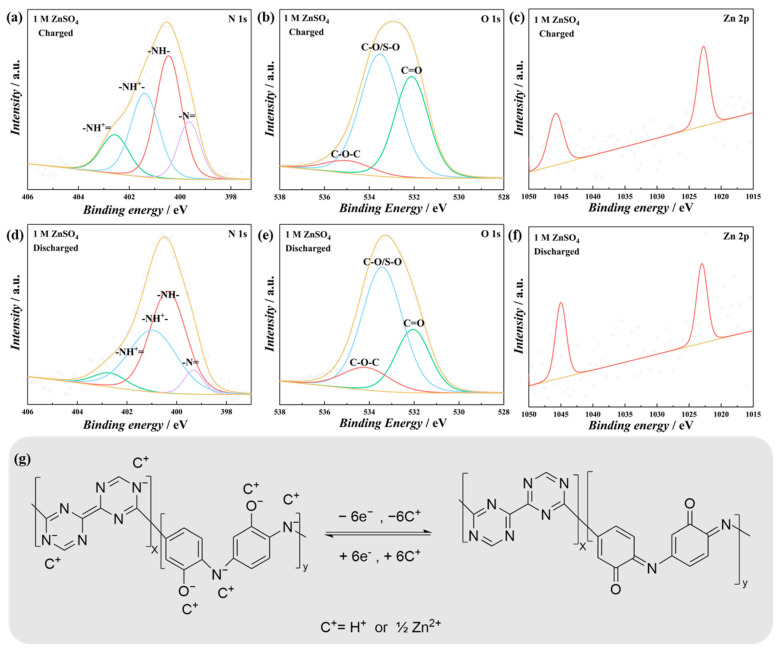
Deconvoluted XPS spectra of PT-*co*-oAP in 1 M ZnSO_4_. (**a**) N 1s region, (**b**) O 1s region, and (**c**) Zn 2p region when charged to 1.1 V; (**d**) N 1s region, (**e**) O 1s region, and (**f**) Zn 2p region when discharged to −0.5 V. (**g**) Proposed charge–discharge mechanism of PT-*co*-oAP.

The charge storage mechanism of the PT-*co*-oAP was further investigated in both 1 M H_2_SO_4_ and 1 M ZnSO_4_ since the copolymer exhibits the highest specific capacity among these electrodes. Ex situ XPS was carried out to investigate the charge storage mechanism [38]. The high-resolution spectra were deconvoluted, and the percentages of each component in the deconvolution high-resolution XPS spectra are provided in the Supporting Information. In 1 M H_2_SO_4_, the imino groups (-N= and -NH^+^=) account for 63.94% of the total N at the charged state. In the discharged state, the content of imino groups decreases to 37.79% (Figure 6a,c). The results indicate that amines are oxidized to produce imines during charging, while the opposite process occurs during discharging [39]. The ratio of C=O to C-O in the deconvolution O 1s spectra also decreases at the discharged state (Figure 6b,d). These observations are consistent with the redox peaks observed in CV. In 1 M ZnSO_4_, the content of imino groups in the N 1s spectra decreases from 29.96% at the charged state to 12.22% at the discharged state. The ratio of C=O to C-O decreases from 0.71 at the charged state to 0.43 at the discharged state. Figure 7c and f show the variation in Zn peak intensity in 1 M ZnSO_4_, which is lower at the charged state and higher at the discharged state. This also indicates that Zn^2+^ is inserted into the polymer at the discharged state. Figure 7g displays the proposed charge storage mechanism of PT-*co*-oAP.

**Figure 8:**
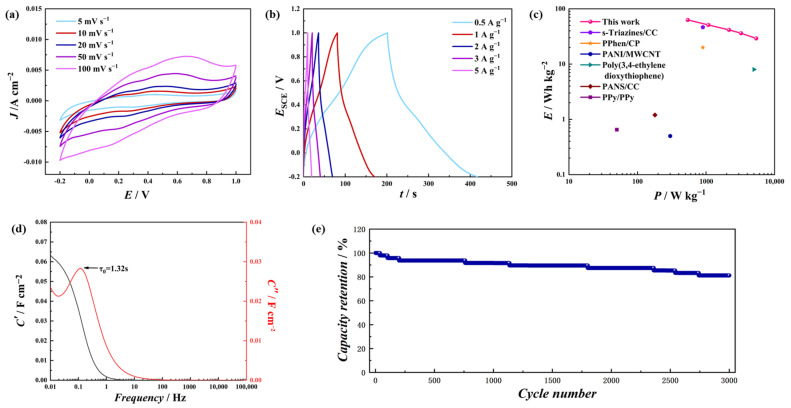
(**a**) CV of the device with 1 M H_2_SO_4_ aqueous electrolyte; (**b**) the GCD profiles of the device at different current densities; (**c**) the Ragone plot comparing the device in this work with systems in the literature based on conducting polymers; (**d**) the dependence of *C*’ and *C*″ over frequency; (**e**) capacity retention versus cycle numbers of the device at 10 A g^−1^.

A two-electrode test was further carried out, with two PT-*co*-oAP electrodes and aqueous 1 M H_2_SO_4_ as the electrolyte, to construct a charge storage device (Figure 8). Figure 8a shows the CV of this device at various scan rates. The CV increases in current density with an increased scan rate, and redox peaks are observed. The GCD curve also shows plateaus, which implies its battery-type behavior [40,41]. The specific capacities at 0.5, 1, 2, 3, and 5 A g^−1^ are 30, 24.9, 18.5, 16.1, and 13.1 mAh g^−1^, respectively (Figure 8b). Figure 8c shows the Ragone plots comparing this device with those reported in the literature [42]. The maximum energy density of the device reaches 63 Wh kg^−1^ at 0.5 A g^−1^. The *C*’ and *C*″ vary with frequency (Figure 8d), and the dielectric relaxation time constant *τ*_0_ of the device was calculated to be 1.32 s. This value is lower than that of some carbon-based supercapacitor electrode materials, which shows its ability to charge and discharge rapidly [43,44]. The stability analysis of the discharging device is performed, as shown in Figure 8e. The device shows high stability (81.2%, 3000 cycles).

## 4. Conclusions

Copolymers of s-triazine and oAP are electropolymerized on the CC substrate using a galvanostatic method. The PT-*co*-oAP exhibits a rough surface and can be used as a battery-type electrode material to electrochemically store charge in both 1 M H_2_SO_4_ and 1 M ZnSO_4_ aqueous solutions. At 1 A g^−1^, the specific capacities of PT-*co*-oAP are 101.3 mAh g^−1^ and 75.0 mAh g^−1^ in 1 M H_2_SO_4_ and in 1 M ZnSO_4_, respectively, higher than those of PT and PoAP. The high specific capacity of PT-*co*-oAP stems from the increased number of active sites and the improved diffusion kinetics, induced by the unique structure owing to the formation of a copolymer. The charge storage mechanism involves the redox processes of amino/imino groups and hydroxyl/carbonyl groups in the copolymer, together with the insertion of cations (Zn^2+^ and/or H^+^). The PT-*co*-oAP exhibits high stability towards GCD cycling. The two-electrode device with PT-*co*-oAP exhibits a 63 Wh kg^−1^ energy density at a 540 W kg^−1^ power density, with 81.2% of the initial specific capacity being maintained after 3000 cycles. This study demonstrates that forming copolymers by electropolymerization is an effective method to improve the specific capacity and energy density of charge storage materials. Further studies focusing on precisely controlling the repeating unit of the polymer by electropolymerization are desirable to achieve high charge storage performance.

## Data Availability

The original contributions presented in this study are included in the article/Supplementary Materials. Further inquiries can be directed to the corresponding author.

## Supplementary Materials

The following supporting information can be downloaded at https://www.mdpi.com/article/10.3390/polym17091160/s1, Figure S1: *E-t* curve for electropolymerization of 5 mM s-triazine and 2 mM oAP in 1 M H_2_SO_4_ at 0.01 A cm^−2^. Working electrode is CC; Figure S2: (a) CV of PT-*co*-oAP at scan rates ranging from 5 to 100 mV s^−1^ in 1 M H_2_SO_4_; (b) Log (*i*, mA) versus (c) log (*v*, mV s^−1^) plots in 1 M H_2_SO_4_.; Figure S3: (a) CV of PT-*co*-oAP at scan rates ranging from 5 to 100 mV s^−1^ in 1 M ZnSO_4_; (b) Log (*i*, mA) versus (c) log (*v*, mV s^−1^) plots in 1 M ZnSO_4_; Figure S4: The cycle stability of these electrodes at 10 A g^−1^ in (a) 1 M H_2_SO_4_ and in (b) 1 M ZnSO_4_; Figure S5: SEM images of the CC substrate; Figure S6: SEM images of the PT-*co*-oAP after GCD cycling at 10 A g^−1^ for 10,000 cycles in 1 M H_2_SO_4_; Figure S7: XPS survey spectrum of PT-*co*-oAP; Figure S8: XPS survey spectrum of PT-*co*-oAP under various conditions. (a) Charged to 1.2 V_SCE_ and (b) discharged to −0.4 V_SCE_ in 1 M H_2_SO_4_; (c) charged to 1.1 V_SCE_ and (d) discharged to −0.5 V_SCE_ in 1 M ZnSO_4_; Figure S9: XPS spectra of (a) C 1s, (b) N 1s, and (c) O 1s regions of PT-*co*-oAP after GCD cycling at 10 A g^−1^ for 10,000 cycles in 1 M H_2_SO_4_. Table S1: Components of deconvoluted C 1s XPS spectra of PT-*co*-oAP in 1 M H_2_SO_4_; Table S2: Components of deconvoluted N 1s XPS spectra of PT-*co*-oAP in 1 M H_2_SO_4_; Table S3: Components of deconvoluted O 1s XPS spectra of PT-*co*-oAP in 1 M H_2_SO_4_; Table S4: Components of deconvoluted N 1s XPS spectra of charged or discharged PT-*co*-oAP in different solutions; Table S5: Components of deconvoluted O 1s XPS spectra of charged or discharged PT-*co*-oAP in different solutions; Table S6: R_CT_ of Nyquist plot in H_2_SO_4_; Table S7: R_CT_ of Nyquist plot in ZnSO_4_; Table S8: Comparison of specific capacity and cyclic stability among some previously reported polymer-based electrochemical energy storage systems. References [9,45,46,47,48,49] are cited in the Supplementary Materials.
