# Supplementary material for: Fabrication of Poly(s-triazine-co-o-aminophenol) Conducting Polymer via Electropolymerization and Its Application in Aqueous Charge Storage
Source: Polymers (Basel). 2025 Apr 24;17(9):1160. doi: 10.3390/polym17091160 (PMC12073347; doi:10.3390/polym17091160)
Supplement: Supplementary file 1 [file polymers-17-01160-s001.zip › polymers-3588479-supplementary.pdf]

Supporting Information

Fabrication of Poly(s-triazine-co-o-aminophenol) Conducting Polymer via Electropolymerization and Its Application in Aqueous Charge Storage

Xueting Bai [1], Bo Lan [1], Xinyang Li [1], Xinlan Yi [2], Shaotong Pei [1] and Chao Wang [3,4,*]

[1] Hebei Provincial Key Laboratory of Power Transmission Equipment Security Defense, North China Electric Power University, Baoding 071003, China; xt_baii@163.com (X.B.); rambo596596@163.com (B.L.); lxygerenyouxiang@163.com (X.L.); peishaotong@ncepu.edu.cn (S.P.)

[2] Economic Management Department, North China Electric Power University, Baoding 071003, China; yixinlan1006@163.com

[3] School of Renewable Energy, Inner Mongolia University of Technology, Ordos 017010, China

[4] Inner Mongolia Key Laboratory of New Energy and Energy Storage Technology, Hohhot 010051, China

* Correspondence: cwang@imut.edu.cn

Table of Contents

1. Experimental Procedure
2. Electrochemistry
3. SEM
4. X-ray photoelectron spectroscopy
5. EIS
6. Comparison of specific capacity and cyclic stability

Experimental Procedure

Chemicals

The following reagents were used without further purification: 1,3,5-triazine ($C_3H_3N_3$, AR, 97%, Shanghai BiDe Pharmaceutical Technology Co., Ltd.), *o*-aminophenol (C_6H_7NO, AR, 99%, Shanghai Aladdin Biochemical Technology Co., Ltd.), potassium ferricyanide ($K_3[Fe(CN)_6]$, AR, 99.5%, Tianjin RuiJinTe Chemicals Co., Ltd.), concentrated sulfuric acid (H_2SO_4, AR, 98.0%, Shanghai HaoHong Bio-Pharmaceutical Technology Co., Ltd.), zinc sulfate ($ZnSO_4$, AR, 99.8%, Shanghai HaoHong Bio-Pharmaceutical Technology Co., Ltd.), carbon cloth (SCC130, Suzhou ShengErNuo Technology Co., Ltd.), and distilled water.

Electrochemical measurements

Electrochemical measurements were carried out at room temperature using the CHI760E electrochemical workstation, and EIS was recorded in the frequency range of 10^6 to 0.01 Hz under a potential amplitude of 5 mV. The specific capacity (C, mAh g^{-1}) was calculated from the discharge branch of the GCD curve using the following formula:

$$C_{S,GCD} = \frac{It}{m} \tag{S1}$$

where I (mA) is the discharge current, t (h) is the discharge time, and m (g) is the mass of the active material on the electrode. The specific capacity (C, mAh g^{-1}) was also calculated based on the integrated charge (Q, C) obtained from the CV cathodic scan using the following formula:

$$C_{S,CV} = \frac{Q}{3.6m} \tag{S2}$$

The capacitance of the two-electrode energy storage system was calculated from the GCD curve using Equation (S3).

$$C_{cell} = \frac{It}{2m \times \Delta V} \tag{S3}$$

where C_{cell}(F g^{-1}) is the specific capacity based on the mass of electrochemically active material, I is the current in A, and ΔV(V) is the potential window.

The energy density and power density of the solid-state supercapacitor were calculated using Equations (S4) and (S5).

$$E = \frac{1}{2 \times 3.6 \times 2m} \int_0^t V dt \tag{S4}$$

$$P = \frac{E \times 3600}{t} \tag{S5}$$

where E ($\mathrm{Wh\,kg^{-1}}$) and P ($\mathrm{W\,kg^{-1}}$) correspond to the energy density and power density, respectively.

Instrumentation

A field emission scanning electron microscope (TESCAN MIRA LMS, Czech Republic) equipped with an electron diffraction spectroscope was used to observe the morphology and elemental distribution of the film. X-ray diffraction (XRD) was performed on an X-ray powder diffractometer (Rigaku SmartLab SE, Japan) equipped with a Cu target (λ = 0.154 nm). X-ray photoelectron spectroscopy (XPS) was carried out using a Shimadzu/Krayos AXIS Ultra DLD at room temperature and ultra-high vacuum (UHV) conditions. The survey spectra were acquired in the binding energy range of 0 – 1200 eV with a step energy of 0.7 eV, a pass energy of 160 eV, and a sweep time of 180 s. The high-resolution spectra were obtained with an energy envelope of 30 eV, a pass energy of 20 eV, a step size of 0.05 eV, and a sweep time of 180 s. No charge correction was applied during the XPS analysis. All high-resolution XPS spectra were calibrated against the C 1 s peak at 284.8 eV when analyzed using the CasaXPS software.

Electrochemistry

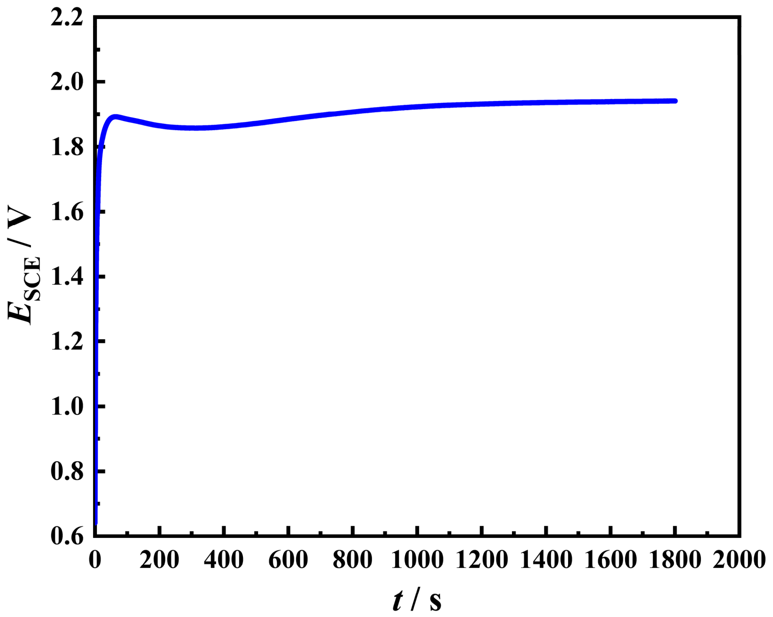

Figure S1. *E* - *t* curve for electropolymerization of 5 mM s-triazine and 2 mM oAP in 1 M H_2SO_4 at 0.01 A cm^{-2}. Working electrode is CC.

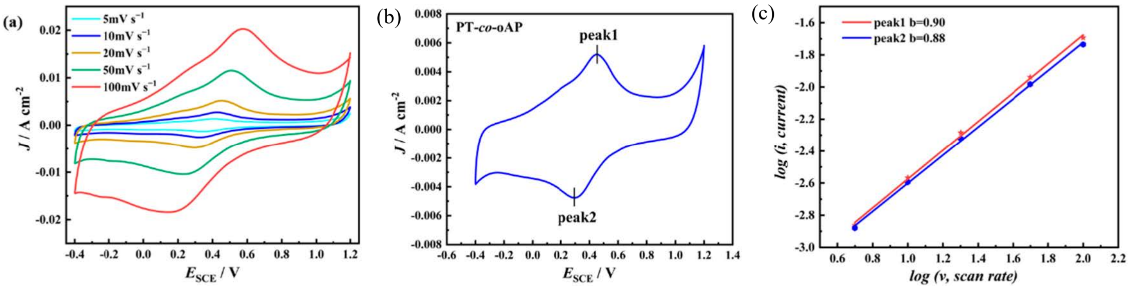

Figure S2. (a) CV of PT-*co*-oAP at scan rates ranging from 5 to 100 mV s^{-1} in 1 M H_2SO_4; (b) Log (*i*, mA) versus (c) log (*v*, mV s^{-1}) plots in 1 M H_2SO_4.

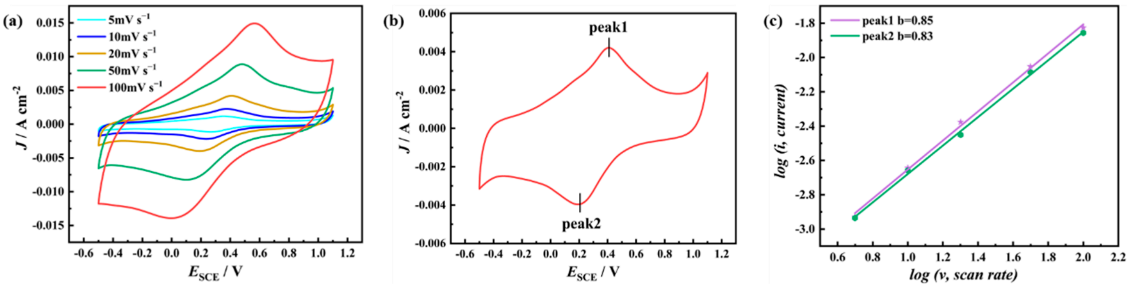

Figure S3. (a) CV of PT-*co*-oAP at scan rates ranging from 5 to 100 mV s^{-1} in 1 M $ZnSO_4$; (b) Log (*i*, mA) versus (c) log (*v*, mV s^{-1}) plots in 1 M $ZnSO_4$.

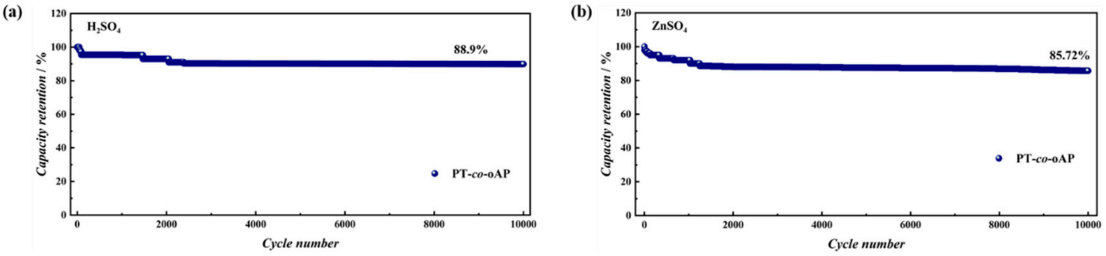

Figure S4. The cycle stability of these electrodes at 10 A g^{-1} in (a) 1 M H_2SO_4 and in (b) 1 M $ZnSO_4$.

SEM

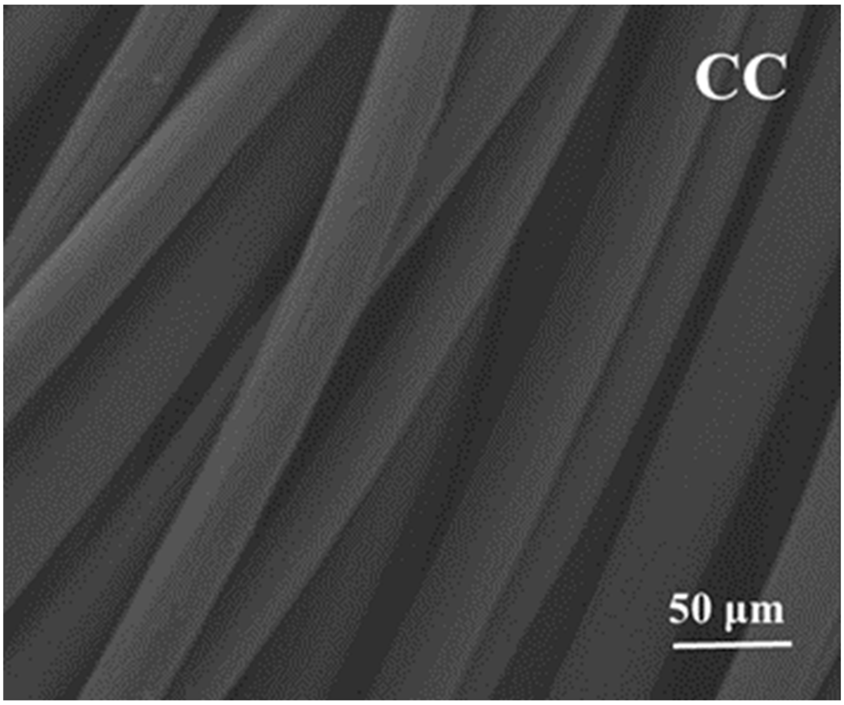

Figure S5. SEM images of the CC substrate.

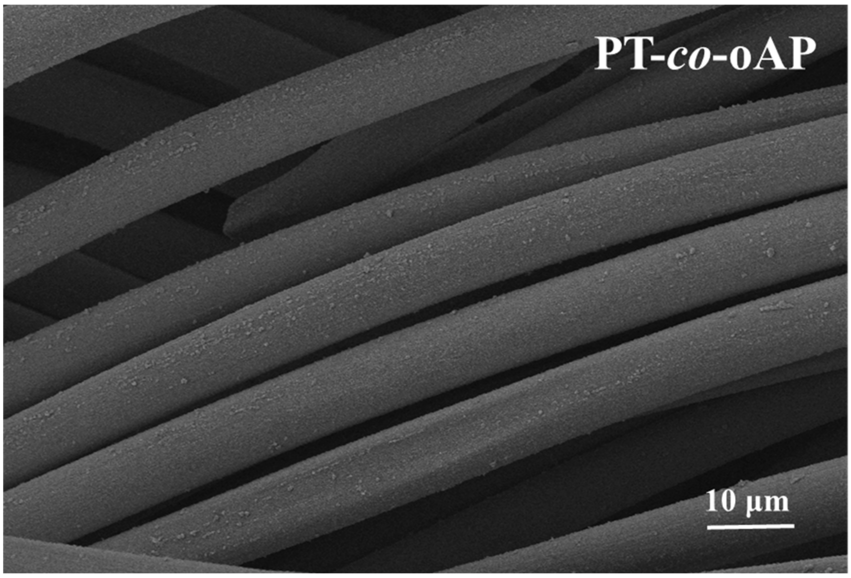

Figure S6. SEM images of the PT-*co*-oAP after GCD cycling at 10 A g^{-1} for 10000 cycles in 1 M H_2SO_4.

X-ray photoelectron spectroscopy

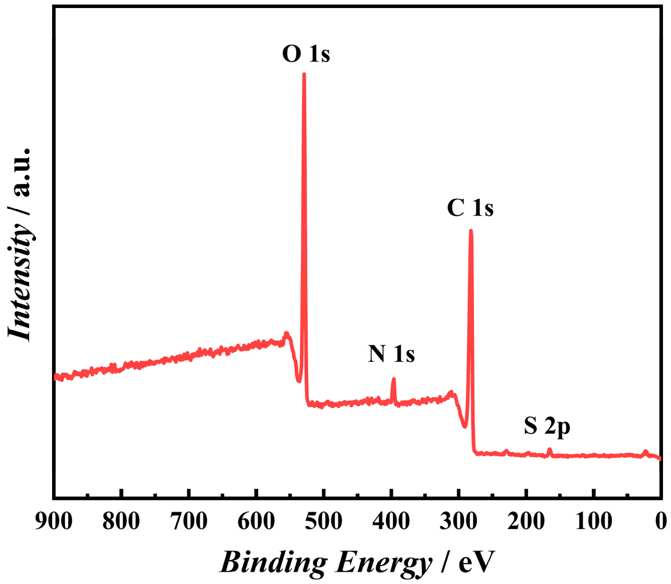

Figure S7. XPS survey spectrum of PT-*co*-oAP.

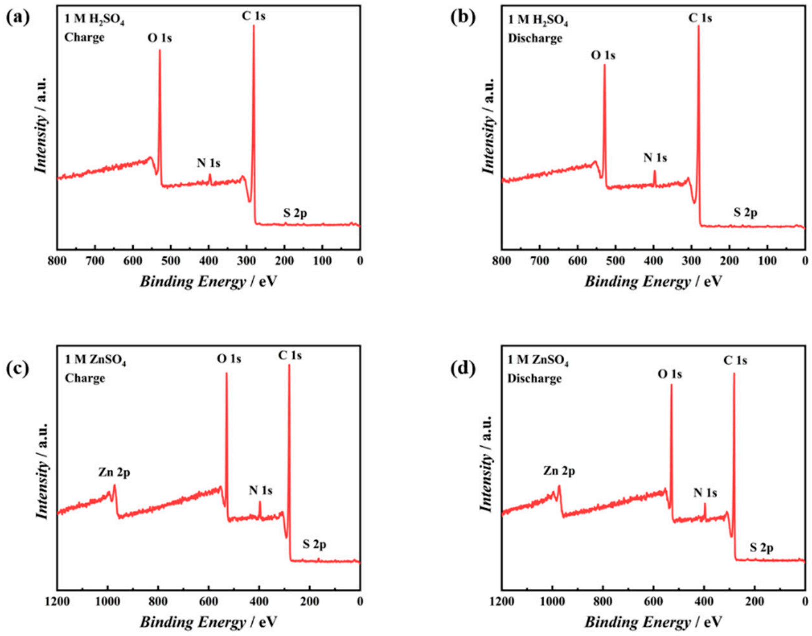

Figure S8. XPS survey spectrum of PT-*co*-oAP under various conditions. (a) Charged to 1.2 V_{SCE} and (b) discharged to -0.4 V_{SCE} in 1 M H_2SO_4; (c) charged to 1.1 V_{SCE} and (d) discharged to -0.5 V_{SCE} in 1 M $ZnSO_4$.

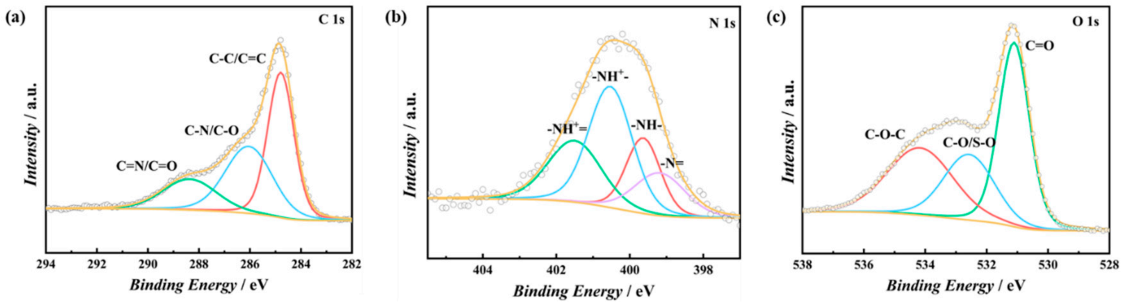

Figure S9. XPS spectra of (a) C 1s, (b) N 1s, and (c) O 1s regions of PT-*co*-oAP after GCD cycling at 10 A g^{-1} for 10000 cycles in 1 M H_2SO_4.

Table S1. Components of deconvoluted C 1s XPS spectra of PT-*co*-oAP in 1 M H_2SO_4.

	284.7 eV<br>C-C/C=C	286.8 eV<br>C-N/C-O	289 eV<br>C=N/C=O
1 M H_2SO_4	48.84	40.55	10.61

Table S2. Components of deconvoluted N 1s XPS spectra of PT-*co*-oAP in 1 M H_2SO_4.

	399.6 eV<br>-N=	400.4 eV<br>-NH-	400.9 eV<br>$-NH^+-$	402 eV<br>$-NH^+=$
1 M H_2SO_4	14.4	31.52	30.69	23.39

Table S3. Components of deconvoluted O 1s XPS spectra of PT-*co*-oAP in 1 M H_2SO_4.

	531.9 eV<br>C=O	533 eV<br>C-O/S-O	534 eV<br>C-O-C
1 M H_2SO_4	32.84	47.32	19.83

Table S4. Components of deconvoluted N 1s XPS spectra of charged or discharged PT-*co*-oAP in different solutions.

	399.6 eV<br>-N=	400.4 eV<br>-NH-	401 eV<br>$-NH^{+}-$	402.6 eV<br>$-NH^{+}=$
1 M H_2SO_4, Charged	38.92	28.19	7.87	25.02
1 M H_2SO_4, Discharged	14.4	31.52	30.69	23.39
1 M $ZnSO_4$, Charged	16.11	40.01	30.04	13.85
1 M $ZnSO_4$, Discharged	6.05	48.5	39.28	6.17

Table S5. Components of deconvoluted O 1s XPS spectra of charged or discharged PT-*co*-oAP in different solutions.

	532.1 eV<br>C=O	533.3 eV<br>C-O/S-O	534.4 eV<br>C-O-C
1 M H_2SO_4, Charged	30.83	50.1	19.07
1 M H_2SO_4, Discharged	19.88	62.95	17.17
1 M $ZnSO_4$, Charged	38.6	54.61	6.8
1 M $ZnSO_4$, Discharged	26.27	60.73	13

EIS

Table S6. R_{CT} of Nyquist plot in H_2SO_4.

**Material**	**R_{CT}(Ω)**
PT	4.84
PT-*co*-oAP	9.47
PoAP	4.05

Table S7. R_{CT} of Nyquist plot in $ZnSO_4$.

**Material**	**R_{CT}(Ω)**
PT	25.84
PT-*co*-oAP	10.23
PoAP	4.03

Comparison of specific capacity and cyclic stability

Table S8. Comparison of specific capacity and cyclic stability among some previously reported polymer-based electrochemical energy storage systems.

Material	Electrolyte	Specific capacity	Cyclic stability	Ref.
PT-*co*-oAP	1 M H_2SO_4	24.9 mAh g^{-1} at 1 A g^{-1}	81.2% after 3000 cycles	This work
s-Triazines/CC	1 M H_2SO_4	93 F g^{-1} at 1 A g^{-1}	93.3% after 2000 cycles	[1]
PPhen/CP	1 M H_2SO_4	401.4 C g^{-1} at 1 A g^{-1}	81.6% after 2000 cycles	[2]
PANI/ MWCNT	PVA/H_2SO_4	180 F g^{-1} at 0.5 A g^{-1}	85.0% after 1500 cycles	[3]
Poly(3,4-ethylenedioxythiophene)	1 M H_2SO_4	86.81 F g^{-1} at 1 mA cm^{-2}	71.6% after 1000 cycles	[4]
PANS/CC	1 M H_2SO_4	307.8 C g^{-1} at 1 A g^{-1}	79.9% after 2500 cycles	[5]
Polypyrrole	PVA/H_2SO_4	23.2 F g^{-1} at 0.5 A g^{-1}	81.0% after 1000 cycles	[6]

References

[1] Pei, S.; Lan, B.; Bai, X.; Liu, Y.; Li, X.; Wang, C. Electropolymerization of s-Triazines and Their Charge Storage Performance in Aqueous Acidic Electrolytes. Polymers 2024, 16, 3266.

[2] Wang, C.; Zhou, Z.; Tian, Q.; Cao, X.; Wu, Y.; Liu, S.; Wang, J. Electropolymerized 1, 10-phenanthroline as the electrode material for aqueous supercapacitors. Chem. Eng. J. 2022, 433, 134483.

[3] Khan, M.Z.; Gul, I.H.; Baig, M.M.; Akram, M.A. Facile synthesis of a multifunctional ternary SnO2/MWCNTs/PANI nanocomposite: detailed analysis of dielectric, electrochemical, and water splitting applications. Electrochim. Acta 2023, 441, 141816.

[4] Lv, T.R.; Zhang, W.H.; Yang, Y.Q.; Zhang, J.C.; Yin, M.J.; Yin, Z.; Yong, K.T.; An, Q.F. Micro/Nano-Fabrication of Flexible Poly (3, 4-Ethylenedioxythiophene)-Based Conductive Films for High-Performance Microdevices. Small 2023, 19, 2301071.

[5] Tian, Y.; Yang, Y.; Wu, Y.; Zhou, Z.; Li, Y.; Wang, J.; Liu, S.; Wang, C. Electropolymerization of 5-amino-2-naphthalenesulfonic acid and their application as the electrode material for supercapacitors. Journal of Energy Storage 2023, 72, 108308.

[6] Zhuo, H.; Hu, Y.; Chen, Z.; Zhong, L. Cellulose carbon aerogel/PPy composites for high-performance supercapacitor. Carbohydr. Polym. 2019, 215, 322-329.
